# Who Benefits Most? Personality Traits as Predictors of Identity Intervention Outcomes in Adolescence

**DOI:** 10.1007/s10964-025-02163-2

**Published:** 2025-03-10

**Authors:** David J. Sandberg, Ann Frisén, Py Liv Eriksson, Moin Syed

**Affiliations:** 1 Department of Psychology, University of Gothenburg, Gothenburg, Sweden; 2 Department of Psychology, University of Minnesota, Minneapolis, MN USA

**Keywords:** Intervention, Personality, Traits, Adolescence, Ethnic-racial identity, The Identity Project

## Abstract

Interventions focused on adolescents’ identity development have shown promising results, but questions remain as to which adolescents benefit most from them. This preregistered study examined how personality traits (Big Five domains and higher-order meta-traits) moderate adolescents’ responsiveness to the Identity Project, a school-based intervention supporting ethnic-racial identity development. A total of 509 adolescents from 22 classrooms in the southwestern regions of Sweden participated in an intervention and control group design (*M*_age_ = 16.28; *SD*_age_ = 0.80; 66% female; 51% migration background). Results indicate that extraversion, a personality trait linked to socialization and external reward-seeking, as well as plasticity, a meta-trait linked to adaptability and exploration, both enhanced adolescents’ responsiveness to the intervention in terms of ethnic-racial identity exploration. Moderation differences were found between genders, but not between migration and non-migration backgrounds. With personality traits and meta-traits being revealed as predictors of intervention effectiveness, the study highlights how not all adolescents benefit equally from interventions targeting identity processes. By adapting interventions like the Identity Project to also reach the introverted or less plastic adolescents, it is possible to make them more inclusive, thus broadening their reach and impact.

## Introduction

Psychosocial interventions designed for adolescents offer opportunities to promote their well-being (Franklin et al., 2017), often doing so by focusing on developmental challenges (Yeager & Walton, 2011). The Identity Project is one such intervention, seeking to encourage adolescents to explore their ethnic-racial identities and to develop a sense of identity resolution (Umaña‐Taylor et al., 2018). While the Identity Project and similar interventions have been linked to decreases in adolescents’ depressive symptoms, increases in academic engagement, and more, there is much that is still unknown about their effectiveness. Are these interventions particularly effective for some adolescents, while others benefit less? One way to understand differences in intervention effectiveness is by examining adolescents’ personality traits, which tend to shape how they engage with their environments (McAdams & Olson, 2010) and even respond to developmental experiences (Soto & Tackett, 2015). Given how interventions like the Identity Project encourage adolescents to engage with peers, reflect on their own journeys, share experiences, and perform homework tasks, it is plausible to also assume that different levels of personality traits could lead to different levels of engagement with aspects of the intervention, and therefore different outcomes. Such information regarding differential effectiveness is important to have when evaluating, adapting, or developing similar intervention programs. Accordingly, this study examines whether personality traits moderate the effectiveness of a school-based intervention program supporting adolescents’ ethnic-racial identity development, while also considering migration background and gender as additional moderators of change.

### Intervening in Adolescent Life

Adolescence is often described as a period of intense development, characterized by physical, social, emotional, and cognitive change (Sawyer et al., 2018). Given the rise of many important and impactful challenges during this developmental stage – such as identity formation, peer pressure, academic stress, and emotional regulation difficulties – interventions aimed at psychosocial processes have become a common, yet often imprecise, approach to supporting adolescents (Das et al., 2016). These psychosocial interventions are often designed to address a wide range of issues, including mental health, behavioral problems, and social skills (Taylor et al., 2017), while a few also more narrowly focus on topics such as identity development (Crocetti, Pagano et al., 2024). Regardless of the area of focus, interventions come in many forms, including individual support interventions, guided peer work, group therapy, family-based interventions, school-based programs delivered by teachers, school-based programs delivered by researchers, and community initiatives (Ali et al., 2015; Caprara et al., 2014; Ruini et al., 2009). While many areas of adolescents’ lives are important, such as the vast role that families play in their identity development, externally introduced interventions most commonly take the form of school-based programs. School-based programs – particularly those facilitated by teachers – are sometimes considered cost-effective due to their accessibility and ability to reach large groups of adolescents in structured environments (Weare & Nind, 2011). The primary aims of school-based intervention programs vary, but tend to focus on the promotion of positive psychosocial development, often achieved by providing adolescents with different sets of skills, such as self-reflection (Gutman & Schoon, 2015).

One of the major reasons why psychosocial interventions are so important during adolescence is that it is also a period when mental health issues tend to emerge (Collishaw et al., 2004). Anxiety, depressive symptoms, and even behavioral disorders frequently manifest during adolescence, and early interventions are believed to be impactful in preventing these issues from becoming more severe over time (Kessler et al., 2012). Thus, by intervening early it may be possible for some intervention programs to mitigate the long-term effects of mental health problems and possibly also impact participants’ overall life chances (Arango et al., 2018). However, the implementation of psychosocial interventions is not without challenges. Interventions are more often than not resource-heavy: They require significant time, financial investment, and trained facilitators to be effective. The inherent complexity of adolescent development also means that any intervention must be carefully tailored (or “adapted”), to fit the needs of the target group they address as well as the context in which they are facilitated (Domenech Rodríguez et al., 2011). Indeed, both contextual and individual factors are likely to influence how adolescents respond to interventions, as exemplified in a large, randomized control trial in which adolescents higher in environmental susceptibility also showed greater increases in psychological well-being and resilience following a school-based intervention (Mertens et al., 2022).

Despite the challenges involved with tailoring interventions to fit the many rather than the few, the potential benefits of psychosocial interventions are not to be underestimated. Effective programs have been associated with improved mental health (Barry et al., 2013), better academic performance (Keogh et al., 2006), social-behavioral adjustment (Harrell et al., 2009), and ethnic-racial identity exploration (Umaña‐Taylor et al., 2018). Such results make them important tools that may even enable smoother transitions into adulthood. So, by addressing issues early on in life, psychosocial interventions can have both long-term promotive and preventive effects, possibly reducing the likelihood of adolescents developing more serious problems later in life.

### Interventions Supporting Identity Development

During adolescence, a period marked by exploration and self-discovery, identity development becomes especially salient (Erikson, 1968). While the term *identity* is complex and may represent different content for different individuals, identity can be understood as the subjective experience of “what makes you, you” (Galliher et al., 2017). Interventions that support the process of identity formation are typically designed to help adolescents navigate their evolving sense of self, striving to promote healthy psychosocial development and lessen the negative mental health effects associated with, for example, identity distress (Samuolis et al., 2015). Meta-analytic findings support the overall effectiveness of such psychosocial interventions. For instance, a recent meta-analysis demonstrated small to moderate effect sizes in enhancing adolescents’ personal and social identities, suggesting that these programs can play a meaningful role in identity development (Crocetti, Pagano et al., 2024). Similarly, interventions aimed at educational and professional identity showed medium effect sizes, indicating effectiveness in helping adolescents align different parts of their future self with their current aspirations (Crocetti, De Lise et al., 2024). Still, while identity development can be divided into distinguishable processes, identity exploration is an undertaking in which the timing and content of exploration can vary between individuals (Schwartz et al., 2014). Such variability makes it especially relevant to understand how interventions focused on identity development may affect adolescents differently, and whether specific factors influence their effectiveness at the individual level.

While identity is a concept that contains many different aspects (Kroger et al., 2010), one particularly relevant aspect within diverse and multicultural societies is *ethnic-racial identity*. Ethnic-racial identity is a multidimensional construct that refers to the part of a person’s identity that encompasses their ethnic, racial, and cultural backgrounds (Phinney et al., 1990), as well as the beliefs and attitudes associated with these aspects of the self (Umaña‐Taylor et al.,^ 2014^). Ethnic-racial identity development is a lifelong process dependent on both introspective exploration and behavioral exploration, and becomes especially central during adolescence (McLean & Syed, 2015; Sokol, 2009). Of great importance is also how ethnic-racial identity development and ethnic-racial identity affect has been linked to positive psychosocial outcomes, such as reduced depressive symptoms and increased self-esteem (Rivas-Drake et al., 2014). All adolescents have an ethnic-racial identity, but the development of these aspects of the self are considered particularly relevant for adolescents with minoritized or migration backgrounds, as they often engage in ethnic-racial identity exploration earlier than their non-migration-background peers (Phinney et al., 1990). This variation in development is likely influenced by differences in cultural socialization (Williams et al., 2020), experiences of discrimination (Yip, 2018) and the need to navigate multiple cultural contexts (Jugert & Titzmann, 2020). Given these differences, adolescents’ migration backgrounds and contexts likely also form how they respond to interventions designed to support ethnic-racial identity development; making them important considerations also when evaluating intervention effectiveness.

The Identity Project is a school-based intervention that seeks to tap into the positive links between identity development and psychosocial health, and consists of eight sessions centered on adolescent ethnic-racial identity exploration (Umaña-Taylor & Douglass, 2017). Adolescents in the intervention are encouraged to engage and learn about their peers, reflect on their own personal journeys, share experiences, and undertake homework tasks that aim to deepen their understanding of their ethnic-racial identities. Previous studies on the Identity Project have primarily examined change in ethnic-racial identity exploration and resolution, as well as positive outcomes related to psychosocial health among the respective target groups (Juang et al., 2023). In this case, *ethnic-racial identity exploration* refers to the process of actively engaging with and reflecting on one’s ethnic-racial background, while *ethnic-racial identity resolution* represents the extent to which individuals have achieved clarity and understanding about their ethnic-racial background and its role in their broader sense of self (Phinney et al., 1990; Umaña-Taylor et al., 2014).

Initial and subsequent implementations of the Identity Project in the U.S. have revealed promising effects on both ethnic-racial identity exploration and resolution (Umaña‐Taylor et al. 2018; Umaña-Taylor et al., 2024), and has since also seen implementations in Europe. When the Identity Project was implemented in Germany, participants in the intervention group cross-sectionally reported higher ethnic-racial identity exploration as well as improvements in critical consciousness and outgroup attitudes, but no significant effects were found for identity resolution (Juang et al., 2020). In a second cohort, no differences in ethnic-racial identity exploration or resolution were found between intervention and control groups. In two other studies that featured the German context, teacher-student relationships as well as classroom cultural diversity climates were found to play important roles in moderating the main intervention effects (Hölscher et al.,^ 2024^; Schachner et al., 2024). Similar to Germany, in Italy, the intervention led to increases in ethnic-racial identity exploration but not resolution (Ceccon et al., 2023). Further analysis showed that adolescents’ ethnic-racial identity resolution did not follow a single pattern of change but instead consisted of multiple change trajectories (Ceccon et al., 2024). In Sweden, studies revealed initial and simultaneous effects of the intervention on ethnic-racial identity exploration and resolution (Abdullahi et al., 2024) but did not show any secondary positive effects on outgroup or diversity attitudes (Sandberg, Frisén et al., 2024). While prior studies have examined how the Identity Project influences ethnic-racial identity exploration, they have primarily assessed exploration as a broad construct rather than distinguishing between introspective exploration (search) and behavioral exploration (participation) components (Syed et al., 2013). Differentiating these processes is however important, as interventions like the Identity Project encourage both reflection (search) and social engagement (participation) to varying degrees (Abdullahi et al., 2024). Taken together, previous studies on the Identity Project intervention have typically shown positive effects on ethnic-racial identity exploration, while findings on identity resolution and broader social outcomes have been more varied. Factors such as classroom climate and teacher-student relationships have been identified as influencing the intervention’s effectiveness, meaning that contextual factors likely also play a role in shaping these outcomes.

Building on these works, an important next step is to further examine how individual differences among adolescents may moderate the effectiveness of the Identity Project intervention in promoting ethnic-racial identity developments; both in the form of ethnic-racial identity exploration (search and participation), and in the form of ethnic-racial identity resolution. This is important not only because interventions like the Identity Project hold promising preventive value; they also offer a means to promote adolescents’ well-being through positive identity development. Still, and as mentioned, these interventions are complex and resource-demanding endeavors, requiring much consideration of their effectiveness and the factors that influence their success. It is therefore important to understand not only whether these interventions work, but also for whom they are most effective (Thomas & Rothman, 2013). Understanding such variations may help researchers adapt identity-based interventions, but also ensure that they benefit as many adolescents as possible. One way to go about this is by looking at adolescents’ personality traits (McCrae & Costa, 1987), and whether such traits moderate the effectiveness of interventions.

### Personality Traits

*Personality traits* are patterns of thoughts, feelings, and behaviors that differ between individuals (McAdams & Olson, 2010). During adolescence, personality traits play an important role in shaping various aspects of life, including social relationships, academic performance, and overall emotional well-being (Soto & Tackett, 2015). Understanding how these traits influence intervention effectiveness, then, is also important for understanding how adolescents engage with developmental challenges, including those posed by interventions such as the Identity Project. While previous studies have examined the relationship between personality and identity development (Klimstra, 2012), it remains unclear how adolescents’ personality traits influence the outcomes of interventions designed to promote identity development. More specifically, there is limited understanding as to whether certain traits either enhance or hinder the effectiveness of these interventions. Conceptually, personality traits and identity both describe aspects of the self, but with different degrees of stability. Personality traits are considered relatively stable, enduring patterns of an individual and their behaviors, while identity is considered more of an ongoing and often shifting process of exploration and commitment, shaped by personal experiences, social interactions, cultural influences, and more.

Personality traits can be examined within a hierarchical model that varies in specificity (DeYoung, 2006), including levels such as the Big Five personality traits (McCrae & Costa, 1987), but also meta-traits (DeYoung, 2015). The Big Five organizes personality traits into five broad domains: openness, conscientiousness, extraversion, agreeableness, and neuroticism. This level of specificity represents a widely used structure of personality traits, with each domain encompassing specific facets or behaviors. The first trait, “openness”, reflects cognitive exploration and a drive for novelty, meaning that individuals high in openness tend to be curious and open-minded while those on the low end prioritize conventionality and familiarity. The second trait, “conscientiousness”, pertains to behavioral regulation in pursuit of goals; here, high levels indicate organization and self-discipline while low levels reflect spontaneity and flexibility. The third trait, “extraversion”, involves reward-seeking through social interaction, with high extraversion marked by sociability and enthusiasm while low extraversion indicates a preference for solitude or lower stimulation. The fourth trait, “agreeableness”, emphasizes cooperative and compassionate behavior; high levels reflect empathy and friendliness while low levels are linked to competitiveness and critical tendencies. Finally, the fifth trait, “neuroticism”, concerns sensitivity to threat and emotional regulation, with high neuroticism reflecting emotional reactivity and low neuroticism being associated with e.g. lower susceptibility to emotional distress. Personality traits can also be approached using a higher-order factor model in which traits are ordered into two, broader, meta-traits of “stability” and “plasticity”, which represent broad dispositional patterns in how individuals interact with the world (DeYoung, 2015). Stability reflects the capacity for self-regulation and maintaining order, encompassing the shared variance among low neuroticism, high agreeableness, and high conscientiousness. Plasticity reflects adaptability and exploration, encompassing the shared variance among high extraversion and high openness.

Personality traits and meta-traits are not only relatively stable individual differences, they also have well-documented gender variations (Feingold, 1994). Research has for example consistently found that girls tend to score higher on agreeableness and neuroticism compared to boys, while boys often exhibit higher assertiveness, a facet of extraversion (Costa et al., 2001). Given these established differences, gender may actually influence how personality traits interact with intervention responsiveness as well. In fact, there is good reason to believe that Big Five traits and meta-traits would be associated with how adolescents respond to an intervention such as the Identity Project.

### Personality Traits and Intervention

While there are both links and overlap between personality traits and identity, most prior research in this conjunction of research topics has focused on changing mean levels and rank-order relations for personality traits (Bleidorn et al., 2021) rather than, on identity intervention susceptibility or the role that personality traits play in identity development. Previous research indicates that personality traits are most relevant for understanding identity, as individuals who are high in openness and conscientiousness often also report positive levels of identity exploration (Tesch & Cameron, 1987). This could be due to individuals high in such traits also tending to describe themselves as more open and thorough when exploring who they are (Hogan & Ones, 1997). In terms of conscientiousness, individuals high in this trait are also more likely to appear more resolute in their identity commitments, hinting at a possible link to factors such as identity resolution (Hirschi, 2012). Extraversion and agreeableness both have interactional qualities, and are likely associated with more socialized aspects of identity development (Han & Pistole, 2017). This is exemplified in another study in which those who were more extraverted than their peers were also more achieved in their identities, and those who were higher in agreeableness were less likely to experience a sense of identity diffusion or loss of direction regarding themselves (Clancy & Dollinger, 1993). Meanwhile, higher neuroticism could be negatively associated with deeper identity exploration, possibly because these individuals may find the process particularly taxing and thus refrain from any excessive exploration (Kawamoto, 2021). Still, and on the contrary, other studies have also found higher neuroticism to be related to the emergence of identity exploration over time (Hirschi, 2012), indicating that personality traits may have different impacts on different levels of identity processes. This relationship between personality traits and identity development could thus also be relevant when examining different types of identity processes; such as ethnic-racial exploration (search), ethnic-racial exploration (participation), and ethnic-racial resolution.

The two meta-traits, plasticity and stability, also have clear relevance for responsiveness to identity interventions. Plasticity involves engaging in exploration of oneself and the surrounding world, whereas stability can indicate a greater sense of rigidity of the self (DeYoung, 2015). As such, this higher-order organization of personality could prove useful when researchers examine ethnic-racial identity development. For instance, plasticity is a meta-trait that is closely aligned with exploration of both the self and the surrounding world, making it potentially influential in ethnic-racial identity exploration. Conversely, stability might reflect a more cautious or reserved approach to such exploration, which could affect the degree to which adolescents engage with identity-related material. Given the overlap (but distinct conceptualizations) between meta-traits and identity exploration, using this higher-order approach also offers a somewhat more parsimonious way to study the interaction between personality and identity development.

Previous studies from a wide array of research fields point toward personality traits also functioning as patterns of characteristics that make individuals more or less susceptible to interventions. Examples of this include how individuals high in agreeableness, conscientiousness, and extraversion seem to benefit more from executive function interventions (Cerino et al., 2020), or how adolescents high in neuroticism and extraversion, and low in conscientiousness, openness, and agreeableness benefited the most from a school-based intervention program promoting positive psychosocial developments (Mertens et al., 2022). In another intervention aimed at fostering self-efficacy, greater benefits were found for those who were high in neuroticism and lower in conscientiousness, agreeableness, and extraversion (Franks et al., 2009). As such, the way in which personality traits moderate intervention effects may largely be a matter of “differential susceptibility”; that is, individual differences in reactivity concerning what is being facilitated through an intervention program. Only one study has so far examined any form of individual-level differential susceptibility related to the Identity Project intervention: one in which participants’ levels of environmental sensitivity were assessed (Ceccon et al., 2023). In this case, environmental sensitivity was defined as an inherited trait that was responsible for individual differences in responses to stimuli (Pluess, 2015), and results suggest that the highly sensitive individuals who participated in the study also saw greater intervention effect in terms of higher levels of ethnic-racial identity exploration. As such, while there is little to no research on how personality traits impact the efficacy of interventions that promote identity development specifically, these previous examples point to the likelihood that intervention effectiveness is also related to personality traits in the field of ethnic-racial identity development. Learning more about the role of personality traits and their impact on the Identity Project intervention can therefore provide insights into why certain adolescents might benefit more also from identity-related interventions. Before turning to the specific aims and research questions, it is important to briefly note the setting in which the research takes place: the sociocultural context of Sweden.

### The Swedish Sociocultural Context

Sweden, a population-wise small country in northern Europe, has undergone significant demographic shifts in recent decades, making it an increasingly ethnoracially diverse society. Today, approximately one in four individuals in Sweden has at least one parent born outside the country, a figure that rises to nearly 40% among adolescents (Statistics Sweden, 2023). While Sweden is often recognized for its tolerant multicultural policies and strong social welfare system, ethnic segregation has been increasing (Hedström, 2019), and attitudes toward immigration have fluctuated, with recent surveys indicating a rise in anti-immigrant sentiments (European Social Survey European Research Infrastructure, 2023). Many adolescents that reside in Sweden, particularly those with migration backgrounds, report experiences of discrimination and social exclusion (Barnombudsmannen, 2021); experiences known also to shape the ways in which identity exploration is understood and approached (Yip, 2018). These sociocultural experiences then create the environment in which ethnic-racial identity development occurs for many adolescents, but also the setting in which the current intervention study was facilitated. The Swedish context itself is thus important in the sense that it is likely to impact how adolescents within Sweden engage (or do not engage) with their ethnic-racial identities.

Another important aspect of the Swedish context to note is the terminology used to discuss matters such as ethnic-racial identity and group belonging. Like in many European countries, the concept of “race” is rarely used in public discourse or research due to its historical association with pseudo-scientific racial classifications (von Brömssen, 2021). Instead, adolescents often self-categorize through nationalities, or are categorized along a Swedish vs. immigrant divide (Svensson & Syed, 2019). At the same time, the word “ethnicity” in Sweden is often defined based on shared national or ethnic origins, skin color, or other distinguishing characteristics, therefore including aspects of social race into the term ethnicity (Diskrimineringsombudsmannen, 2024). This ambiguity of different ethnoracial concepts, combined with a social tendency toward colorblindness, could be one of the reasons why both adolescents and adults in the Swedish context sometimes find it difficult to approach these topics (Sandberg, Berne et al., 2024). To summarize, adolescents in Sweden likely explore their ethnic-racial identities within a context where belonging and marginalization are both common experiences, something that will eventually shape their ethnic-racial identity journeys. These contextual factors provide an important backdrop for understanding how personality traits may influence the effectiveness of the Identity Project in promoting ethnic-racial identity development.

## Current Study

There is much value in understanding if supporting adolescents’ identity development through interventions is effective for the many rather than the few. The current study focuses on an intervention designed to promote ethnic-racial identity development and whether personality traits impact its effectiveness. Through this approach, the knowledge gained will not only fill a gap in researchers’ understanding of intervention efficacy, but may also further their ability to adapt identity-related interventions, shaping them to be more accessible to all adolescents, regardless of their personalities. The current, preregistered study (https://osf.io/j4baq) thus aims to investigate whether personality traits impact the efficacy of a school-based intervention focused on ethnic-racial identity development. It does this through a wait-list control study design with three measurements spanning 16 weeks. A total of four research questions were formulated based on prior research. The first question examined whether baseline mean scores on the personality traits “openness”, “conscientiousness”, “extraversion”, “agreeableness”, and “neuroticism” predict changes in ethnic-racial identity exploration and resolution in the intervention group relative to the control group, from T1 to T3. The second question examined whether aggregated baseline scores on the meta-traits “plasticity” and “stability” predict changes in ethnic-racial identity exploration and resolution in the intervention group relative to the control group, from T1 to T3. Due to the impact of migration backgrounds on ethnic-racial identity development, as well as the gendered differences in personality traits, two more research questions were formulated. The third question examined whether adolescents’ migration backgrounds moderate the association between personality traits, meta-traits, and ethnic-racial identity intervention effect. The fourth question examined whether adolescents’ self-reported gender moderates the association between personality traits, meta-traits, and ethnic-racial identity intervention effect.

## Method

### Participants

Participants from 22 tenth-grade classrooms took part in the intervention study as well as four waves of survey measurements. All participants attended one of four high schools in the Västra Götaland region of Sweden. The schools’ sociodemographic information was assessed prior to recruitment in order to involve participants and classrooms with ethnoracial heterogeneity, using the SIRIS database (Swedish National Agency for Education, 2020). From the 22 classrooms – comprising students of natural sciences, economics, agriculture, social sciences, craftmanship, childcare, caregiving, and individually adapted programs – a total of 509 adolescents participated in the study (*M*_age_ = 16.28; *SD*_age_ = 0.80; *M*_range_ = 15–19; 66% female; 51% migration background). Participants were categorized based on migration background, using self-reported information on whether they or their parents were born outside of Sweden. Based on these responses, 52% (*n* = 266) were classified as having a migration background, while 40% (*n* = 205) were classified as non-migration background (born in Sweden with at least one parent also born in Sweden). An additional 8% (*n* = 43) had missing data on this item due to non-response or absence. Half of the classes from each school were assigned to the experiment group and the other half to the wait-list control group, through randomization. While the participants took part in four measurements in total during the fall semester of 2021 and the spring semester of 2022, only data from the first three time points are used in this study due to the fourth not being a true control time point (the control group had also received the intervention by then).

Regarding potential differences between groupings, the intervention and control groups did not differ in terms of conditions or main variables of interest – migration background (*χ*^*2*^(1) = 1.95, *p* = 0.21); gender (*χ*^*2*^(2) = 0.71, *p* = 0.70); T1 ethnic-racial identity resolution scores (*t*(432) = −0.36, *p* = 0.72); or T1 personality scores (*t*_*openness*_(408) = 1.23, *p* = 0.22; *t*_*conscientiousness*_(371) = −0.84, *p* = 0.40; *t*_*extraversion*_ (385) = −0.04, *p* = 0.97; *t*_*agreeableness*_(389) = 0.44, *p* = 0.66; *t*_*neuroticism*_(361) = 0.88, *p* = 0.38) – but did differ in terms of T1 ethnic-racial identity exploration scores (*t*(432) = 2.84, *p* = 0.005). The overall attrition rate of the sample was moderate to high, with 39% of adolescents having missing data for at least one measurement. Subsequent participant retention rates were strong over time (T2 = 84.7%; T3 = 84.9%; T4 = 80.0%), and few adolescents missed more than one measurement. Attrition across time and dropouts were not associated with participants’ gender (*χ*^*2*^(2) = 0.58, *p* = 0.75), migration background (*χ*^*2*^(2) = 1.05, *p* = 0.31), T1 ethnic-racial identity exploration scores (*p* = 0.20), or T1 personality traits (*p*_*openness*_ = 0.76; *p*_*conscientiousness*_ = 0.29; *p*_*extraversion*_ = 0.81; *p*_*agreeableness*_ = 0.55; *p*_*neuroticism*_ = 0.34). Participants with missing data for any reason other than canceling participation were included in the dataset using a Full Information Maximum Likelihood (FIML) approach to account for the missingness. This means that even adolescents who were missing at baseline were still included in the study sample.

### Procedure

To recruit participants for the study, a list of high schools in two cities located in the region of Västra Götaland, Sweden, was first compiled. High schools were considered eligible for inclusion if, according to statistics in the SIRIS database, official records of the Swedish National Agency for Education (2020), at least 40% of their students were identified as having non-Swedish backgrounds. Principals from 19 high schools that fulfilled these criteria were contacted via email with study information, followed by a follow-up phone call. Of these, eight schools declined participation, and seven did not respond. For the schools that expressed interest in participating, meetings were arranged to provide principals and teachers the opportunity to ask clarifying questions about the intervention. The four remaining schools were then formally invited and agreed to participate in the study. Research team members, along with teachers, visited all of the 22 participating classrooms to inform students about the upcoming intervention, information that was also provided to students in written form. All schools agreed to implement the eight-session intervention during regular school hours, integrating them into existing class schedules (most commonly during social sciences lessons, instead of adding extra class time for the students). For more detail on the intervention’s content and eight-week curriculum, please see the available supplementary material on the Open Science Framework platform (https://osf.io/dknhs).

Data collection occurred at four points in time, T1 through T4, the first three of which were used in the current study. At each of these times students completed online questionnaires, presented in Swedish, administered via the Qualtrics platform. They completed the questionnaires on either their mobile phones or school-issued computers, remaining in the classroom for the duration. They were provided with a snack while completing the survey, but received no other compensation. The intervention group participated in the Identity Project between T1 (baseline) and T2 (12 weeks from baseline), while the wait-list control group received the intervention after T3 (16 weeks from baseline) and completed their final questionnaire at T4 (26 weeks from baseline). For the questionnaires, professional translations were done when no Swedish versions were available. To ensure accuracy, the translated versions were back translated into English and compared to the original scales. Following these translations, a pilot test was conducted with 30 high school students who were asked to flag any questions or wording they found difficult and to provide general feedback on the questionnaires. Minor adjustments to the wording were made based on this pilot feedback. To further assist students during the measurement sessions, a glossary explaining difficult terms was also distributed with the questionnaires. During each data collection session one to three moderators, and often a teacher, were present in the classroom to assist with questions or provide language support. All moderators were trained clinical psychologists who were actively involved in the research project. In all classrooms but one, the moderators were paired to ensure representation of both majoritized and minoritized ethnoracial backgrounds within the classrooms.

### Measures

#### Personality Traits

Traits were measured using the Big Five Inventory scale – BFI (John et al., 1991) at T1 only. The scale has previously been validated for use in the Swedish context, but not with adolescents. The BFI includes a total of 44 items and consists of five subscales: openness, conscientiousness, extraversion, agreeableness, and neuroticism. Example items include “I see myself as someone who is talkative” and “I get nervous easily”. Items are answered on a five-point Likert scale ranging from (1) Does not describe me at all to (5) Describes me very well. This same scale was used to examine the higher-order meta-traits plasticity and stability, which are different combinations of the five subscales grounded in the mutual explained variance between these traits (DeYoung, 2006). Plasticity is statistically aggregated from the shared variance between openness and extraversion, while stability is aggregated from the shared variance between conscientiousness, agreeableness, and reversed neuroticism (emotional stability). The Openness subscale demonstrated moderate internal consistency (*α* = 0.60), the Conscientiousness subscale acceptable internal consistency (*α* = 0.73), the Extraversion subscale acceptable internal consistency (*α* = 0.70), the Agreeableness subscale lower internal consistency (*α* = 0.59), and the Neuroticism subscale acceptable internal consistency (*α* = 0.76).

#### Ethnic-Racial Identity Exploration

Exploration was measured using two scales as they capture slightly different aspects of ethnic-racial identity exploration: exploration (participation) and exploration (search). Exploration (participation) was measured using the exploration subscale of the Ethnic Identity Scale – Brief, EIS-B (Douglass & Umaña-Taylor, 2015). The items in the EIS-B scale focus on participatory aspects of ethnic-racial identity exploration, e.g. “I have attended events that have helped me learn more about my ethnicity”. The subscale includes three items and is measured using a four-point Likert scale ranging from (1) Does not describe me at all to (4) Describes me very well. Internal consistency of the EIS-B exploration scale was very good (*α*_T1_ = 0.80; *α*_T2_ = 0.82; *α*_T3_ = 0.85; *α*_T4_ = 0.85). Exploration (search) was measured using the Multigroup Ethnic Identity Measure, MEIM (Phinney, 1992). The items in the MEIM scale focus on introspective aspects of ethnic-racial identity exploration, e.g. “I think a lot about how my life will be affected by my ethnic group membership”. The subscale includes five items and is measured using a four-point Likert scale ranging from (1) Strongly disagree to (4) Strongly agree. Internal consistency of the MEIM was good (*α*_T1_ = 0.69; *α*_T2_ = 0.76; *α*_T3_ = 0.79; *α*_T4_ = 0.80).

#### Ethnic-Racial Identity Resolution

Resolution was measured using the resolution subscale of the Ethnic Identity Scale – Brief, EIS-B (Douglass & Umaña-Taylor, 2015). The subscale includes three items, e.g. “I have a clear sense of what my ethnicity means to me,” and is measured using a four-point Likert scale ranging from (1) Does not describe me at all to (4) Describes me very well. Internal consistency of the EIS-B resolution scale was very good (*α*_T1_ = 0.84; *α*_T2_ = 0.84; *α*_T3_ = 0.85; *α*_T4_ = 0.88).

#### Migration Background

Adolescents’ migration backgrounds were dichotomized using self-reports of their own and their parents’ birth countries. In line with how the Swedish National Statistics Bureau categorizes background information (Statistics Sweden, 2002), adolescents with two parents born abroad, regardless of birthplace, were coded as having migration backgrounds. Adolescents with one or more parents born in Sweden, regardless of birthplace, were coded as having non-migration backgrounds. In cases in which at least one parent’s birth country was missing and the adolescent was born in Sweden, the adolescent was coded as having a non-migration background. In cases in which at least one parent’s birth country was missing and the adolescent was born outside Sweden, the adolescent was coded as having a migration background.

#### Gender

Answered by participants at baseline in the form of one statement with three possible answers: “I identify as a” (1) Boy, (2) Girl, or (3) That division does not suit me.

### Analytic Strategy

The analyses that are part of this study were preregistered on the Open Science Framework (https://osf.io/j4baq). One deviation from the preregistration was made: the exclusion of an exploratory research question focused on the relations and change between all ethnic-racial identity measures conjointly (noted as “research question 5” on the preregistration form). This decision was made based on the insufficient change in two out of three of the included dependent variables, leading to poor model fit and lacking predictive quality when all variables were included as interaction terms within a large model. All other reported analyses were preregistered.

**Fig. 1:**
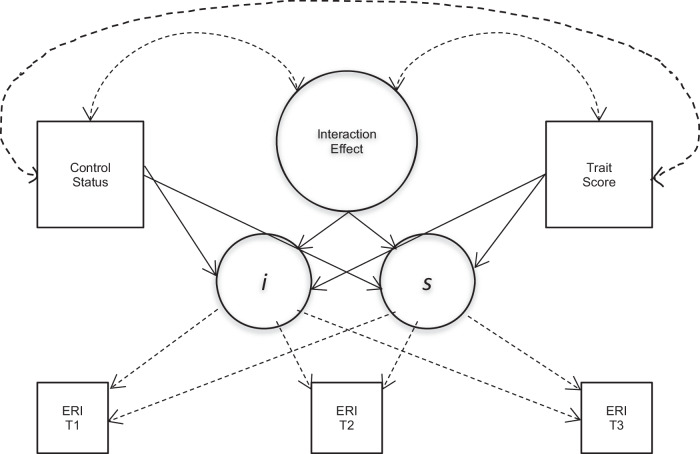
Latent Growth Curve Model (LGCM) used to assess the interaction effect of personality traits and intervention status on ethnic-racial identity growth (‘ERI’ in the figure). Note. Used to test research questions 1 and 2. *i* = linear intercept estimates, *s* = linear slope estimates

Descriptive statistics, histograms, and boxplots were examined to screen for distribution and any patterns or outliers in the dataset. A structural equation modeling (SEM) framework and primarily latent growth curve models (LGCMs) in the *lavaan* package of R-statistics (Rosseel, 2012) were used to assess mean-level change and interactions between all dependent and independent study variables. Interaction terms were created by multiplying the binary and continuous variables of interest (control group status, gender, migration background, and personality trait scores) to test their combined effects on the latent growth parameters (intercepts and slopes). These interaction effects were then specified as predictors within the latent growth models to assess moderation effects on ethnic-racial identity development. The latent growth parameters, means, and standard errors for linear models were examined and used to test the significance of ethnic-racial identity growth in the respective models. Time was modeled in full weeks, with T1 at 0 weeks, T2 at 12 weeks, and T3 at 16 weeks. Fixed slope factor loadings (0/12/16) were used to incorporate time into the latent growth models, with centering at baseline (T1) where the slope loading was set to 0. Unconditional models were run first, followed by the addition of intervention group status, and finally the moderating variables. Random intercepts and slopes were estimated to account for individual differences in baseline levels and growth trajectories. The effects of control group status, personality traits, migration background, gender, and their respective interactions, were specified as fixed to ensure model stability while still capturing overall patterns of change. Fit statistics such as the comparative fit index (CFI), the root mean square error of approximation (RMSEA), and the sample size-adjusted Bayesian information criterion (ssBIC) were assessed to determine model fit. Personality traits and meta-traits were run in separate models to avoid the multicollinearity issues caused by interaction effects, often comprising the same variables at multiple times. As it proved challenging to interpret the three-way interaction effects and their directions, personality scores were mean centered throughout, and “low” or “high” personality trait levels were created using −1 and +1 standard deviations (for plotting purposes only). All data analyses and models were run and fit in RStudio Build 576 (R Core Team, 2021; RStudio Team, 2020), and inferences were drawn for all parametric analyses at *p* < 0.05. While many models were run for the respective personality traits, all modeling followed similar patterns. First, two-way interaction latent growth curve models were used to assess growth and to test research questions 1 and 2 (Fig. 1).

Following these initial models, higher-level, three-way interaction models were added on top of the previous models and used to test research questions 3 and 4. This moderating layer was entered into the model and allowed for the examination of gender and migration background effect on the personality trait impact of the intervention effect. While gender was included as a moderator in some models, the small number of participants who identified as non-binary (*n* = 6, or 1.2% of the sample) resulted in insufficient statistical power to analyze them as a separate category. Therefore, in any hypothesis testing where gender was included as a moderator, these six individuals were treated as missing for that specific analysis. However, their data remained included in all other relevant analyses. Through this approach with different levels of interactions, all variable main effects are accounted for in the different models, while still allowing the models to assess the impact of personality traits on the effect of the intervention specifically, i.e. the interaction effect. All reported beta coefficients (*b*) are unstandardized estimates, representing the raw change in the latent ethnic-racial identity variable over time per unit change in the predictor. The intercept (*i*) reflects the starting level of ethnic-racial identity at baseline, while the slope (*s*) represents the rate of change over time. The effects presented throughout the Results section specifically refer to changes in the latent slope (*s*) of ethnic-racial identity growth, not the intercept (*i*).

## Results

### Preliminary Analyses

Several preliminary analyses were conducted to evaluate the quality and structure of the dataset. Analyses of intraclass correlation coefficients from a prior study (Sandberg, Frisén et al., 2024) revealed sufficient variability in adolescents’ scores on identity development over time (*r*_explorationEIS_ = 0.43; *r*_explorationMEIM_ = 0.48; *r*_resolution_ = 0.48). Minimal variability was observed at the classroom level (*r*_explorationEIS_ = 0.02; *r*_explorationMEIM_ = 0.06; *r*_resolution_ = 0.03) and at the school level (*r*_explorationEIS_ = 0.05; *r*_explorationMEIM_ = 0.09; *r*_resolution_ = 0.11). Previous analyses of the scales, conducted as part of the main effect evaluation, demonstrated scalar invariance over time for all but one scale, the Multigroup Ethnic Identity Measure (Abdullahi et al., 2024). While this deviation was noted, the differences were not substantial enough to affect the modeling process or compromise the reliability of the results. As such, they were excluded from further consideration in the present study. To assess power within the models, Monte Carlo power analyses using 1000 replications were run. These analyses indicated that the study is well-powered to detect both growth and interaction effects, with estimated power ranging between 95–99%, even when assuming small effect sizes (*N* = 509; β = 0.02). This sensitivity to detect interaction effects was particularly strong when ethnic-racial identity exploration (participation) was used as the dependent variable.

Visual inspection of outliers using boxplots and histograms revealed normal-shaped distribution for all main variables and a reasonable spread in response variability across the dataset. Overall, the dataset showed some expected outliers, but no excessive inconsistencies that could compromise the data quality. The three ethnic-racial identity constructs – exploration (participation), exploration (search), and resolution – were all moderately correlated in positive directions, but distinct enough to be treated as separate constructs within the models (Table 1).

**Table 1:** Means, standard deviations, and bivariate correlations for all parametric study variables

Variable	*M*	*SD*	1	2	3	4	5	6	7	8	9	10	11	12	13	14
1. Openness	3.35	0.53														
2. Conscientiousness	3.30	0.62	0.10*													
3. Extraversion	3.29	0.64	0.34**	0.34**												
4. Agreeableness	3.60	0.53	0.19**	0.31**	0.24**											
5. Neuroticism	3.04	0.74	0.01	0.39**	0.27**	0.15**										
6. Participation T1	2.40	0.87	0.12*	0.14**	0.00	0.03	0.12*									
7. Resolution T1	3.20	0.67	0.10*	0.19**	0.13**	0.13**	0.22**	0.48**								
8. Search T1	2.60	0.66	0.16**	0.15**	0.04	0.03	0.07	0.43**	0.29**							
9. Participation T2	2.67	0.78	0.13*	0.14**	0.03	0.08	0.21**	0.47**	0.30**	0.38**						
10. Resolution T2	3.24	0.64	0.10	0.13*	0.15**	0.18**	0.12*	0.28**	0.47**	0.24**	0.50**					
11. Search T2	2.64	0.67	0.13*	0.14**	0.07	0.01	0.08	0.35**	0.25**	0.47**	0.44**	0.29**				
12. Participation T3	2.74	0.81	0.15**	0.16**	0.12*	0.03	0.08	0.40**	0.28**	0.40**	0.55**	0.29**	0.46**			
13. Resolution T3	3.27	0.66	0.13**	0.25**	0.16**	0.22**	0.18**	0.30**	0.46**	0.32**	0.36**	0.55**	0.30**	0.55**		
14. Search T3	2.75	0.68	0.19**	0.17**	0.14**	0.07	0.06	0.42**	0.31**	0.50**	0.42**	0.33**	0.49**	0.50**	0.33**	

M and SD are used to represent mean and standard deviation. All correlations were mapped using Pearson’s correlation coefficient. N_range_ = 313–454. “Participation” represents ethnic-racial identity exploration (participation), “Search” represents ethnic-racial identity exploration (search), and “Resolution” represents ethnic-racial identity resolution

*p < 0.05, **p < 0.01

Auto-correlations for all ethnic-racial identity measures ranged between 0.40 and 0.55, indicating moderate consistency for the measures between the three time points. To examine potential baseline group differences, various comparison tests were conducted. Previously reported t-tests (Sandberg, Frisén et al., 2024) showed some baseline differences in ethnic-racial identity development by control/experiment group (*p*_explorationEIS_ = <0.01; *p*_explorationMEIM_ = 0.12; *p*_resolution_ = 0.72) and migration background (*p*_explorationEIS_ = <0.01; *p*_explorationMEIM_ = <0.01; *p*_resolution_ = <0.01), indicating that participants in the control group and those with migration backgrounds had already engaged slightly more in ethnic-racial identity exploration comparatively. One-way ANOVAs found minor differences in ethnic-racial identity resolution by gender (*p*_explorationEIS_ = 0.66; *p*_explorationMEIM_ = 0.88; *p*_resolution_ = 0.03) as well as school (*p*_explorationEIS_ = <0.01; *p*_explorationMEIM_ = <0.01; *p*_resolution_ = <0.01) and classroom (*p*_explorationEIS_ = <0.01; *p*_explorationMEIM_ = <0.01; *p*_resolution_ = <0.01), indicating minor differences in levels of ethnic-racial identity measures at baseline. Tukey’s post hoc tests revealed that while significant, the school and classroom differences were minor and limited to comparisons between cities, rather than within-city classroom differences. However, these city-based differences were not explicitly accounted for in the hypothesis testing models. The absence of covariates for classrooms or schools was justified based on two key factors: (1) the need to prioritize parsimony in already complex models, and (2) previously published intraclass correlation coefficient results and baseline difference testing which indicated sufficient control for these variables within the sample (Sandberg, Frisén et al., 2024).

### Personality Traits as Predictors of Ethnic-Racial Identity Change

Main effects and interaction effects were analyzed to determine the role of personality traits in predicting ethnic-racial identity development over time. Extraversion did not have a main effect on ethnic-racial identity exploration (participation) for the whole sample (*b* = −0.006, *p* = 0.33, *β* = −0.116). However, a significant interaction effect was found between extraversion and the intervention, with higher scores on extraversion positively predicting ethnic-racial identity exploration (participation) for the intervention group relative to the control group from T1 to T3 (*b* = 0.027, *p* = 0.001, *β* = 0.402). None of the other four personality traits showed any significant main effects on ethnic-racial identity exploration (participation): openness (*b* = −0.001, *p* = 0.89), conscientiousness (*b* = −0.001, *p* = 0.88), agreeableness (*b* = 0.009, *p* = 0.22), and neuroticism (*b* = −0.002, *p* = 0.67). Similarly, no significant interaction effects were found between the intervention and the other four personality traits in predicting ethnic-racial identity (participation): openness (*b* = 0.006, *p* = 0.60), conscientiousness (*b* = 0.001, *p* = 0.92), agreeableness (*b* = 0.001, *p* = 0.96), and neuroticism (*b* = 0.007, *p* = 0.34).

None of the five personality traits demonstrated significant main effects on ethnic-racial identity exploration (search) over time: openness (*b* = 0.010, *p* = 0.09), conscientiousness (*b* = 0.001*, p* = 0.78), extraversion (*b* = 0.002, *p* = 0.63), agreeableness (*b* = 0.001, *p* = 0.93), and neuroticism (*b* = −0.003, *p* = 0.49). Similarly, no significant interaction effects were found between the intervention and any of the personality traits in predicting ethnic-racial identity exploration (search): openness (*b* = −0.015, *p* = 0.06), conscientiousness (*b* = −0.001, *p* = 0.86), extraversion (*b* = 0.006*, p* = 0.38), agreeableness (*b* = 0.002*, p* = 0.79), and neuroticism (*b* = 0.007, *p* = 0.27).

None of the five personality traits showed significant main effects on ethnic-racial identity resolution over time: openness (*b* = 0.005*, p* = 0.49), conscientiousness (*b* = 0.004*, p* = 0.29), extraversion (*b* = 0.000, *p* = 0.99), agreeableness (*b* = 0.001, *p* = 0.87), and neuroticism (*b* = 0.001, *p* = 0.89). No significant interaction effects were found between the intervention and personality traits in predicting ethnic-racial identity resolution: openness (*b* = −0.005, *p* = 0.58), conscientiousness (*b* = −0.007, *p* = 0.24), extraversion (*b* = 0.005, *p* = 0.48), agreeableness (*b* = 0.008, *p* = 0.34), and neuroticism (*b* = 0.007, *p* = 0.21).

**Fig. 2:**
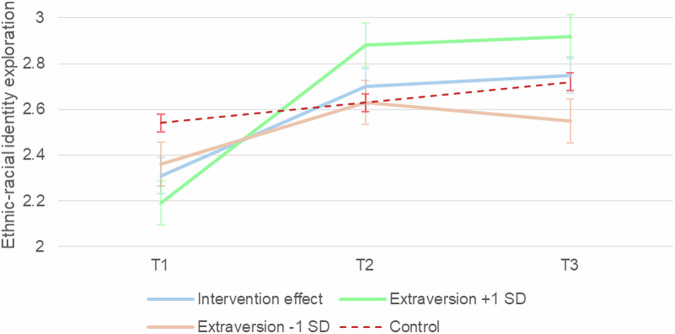
Ethnic-racial identity exploration trajectories by extraversion level within the intervention group. The figure displays raw means and error bars at each time point for participants at ± 1 SD of extraversion, alongside the overall intervention effect and control group trajectory. T1 = baseline, T2 = 12 weeks, T3 = 16 weeks

In summary, extraversion emerged as a key predictor of the intervention’s effectiveness on ethnic-racial identity exploration (participation), meaning that those who were high in extraversion also benefited more in terms of intervention main effect, whereas those who were low in extraversion benefited less (Fig. 2).

### Meta-Traits as Predictors of Ethnic-Racial Identity Change

The meta-trait plasticity did not have a main effect on ethnic-racial identity exploration (participation) for the whole sample (*b* = −0.009, *p* = 0.33, *β* = −0.147). However, a significant interaction effect was found between plasticity and the intervention, with scores on plasticity positively predicting ethnic-racial identity exploration (participation) for the intervention group relative to the control group from T1 to T3 (*b* = 0.033, *p* = 0.008, *β* = 0.360). While plasticity consists of the average scores from both the extraversion and openness traits, this combined higher-order meta-trait fit the data better and explained more of the variance than the Big Five model did (*CFI* = 0.987; *RMSEA* = 0.038; *BIC* = 2671). The meta-trait stability – representing the average scores on the traits conscientiousness, agreeableness, and reversed neuroticism (emotional stability) – did not demonstrate any significant main effects on ethnic-racial identity exploration (participation) over time (*b* = 0.005, *p* = 0.50). Similarly, no significant interaction effects were found between stability and the intervention in predicting ethnic-racial identity exploration (participation) (*b* = −0.006, *p* = 0.62).

Neither of the two meta-traits demonstrated significant main effects on ethnic-racial identity exploration (search) over time: plasticity (*b* = 0.007, *p* = 0.24), stability (*b* = 0.003*, p* = 0.61). Similarly, no significant interaction effects were found between the intervention and either of the meta-traits in predicting ethnic-racial identity exploration (search): plasticity (*b* = −0.004, *p* = 0.62), stability (*b* = −0.007, *p* = 0.45). Neither of the meta-traits demonstrated significant main effects on ethnic-racial identity resolution over time: plasticity (*b* = 0.002, *p* = 0.71), stability (*b* = 0.002*, p* = 0.67). Similarly, no significant interaction effects were found between the intervention and either of the meta-traits in predicting ethnic-racial identity resolution: plasticity (*b* = 0.001, *p* = 0.86), stability (*b* = −0.007, *p* = 0.40).

**Fig. 3:**
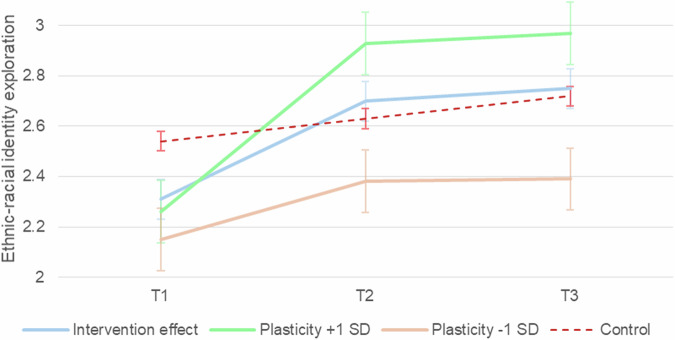
Ethnic-racial identity exploration trajectories by plasticity level within the intervention group. The figure displays raw means and error bars at each time point for participants at ± 1 SD of plasticity, alongside the overall intervention effect and control group trajectory. T1 = baseline, T2 = 12 weeks, T3 = 16 weeks

In summary, plasticity emerged as a key predictor of the intervention’s effectiveness on ethnic-racial identity exploration (participation), meaning that those who were high in plasticity also benefited more in terms of intervention main effect, whereas those who were low in plasticity benefited less (Fig. 3).

### Migration Background as a Moderator of Personality Trait Influence on Intervention Efficacy

Three-way interaction effects between migration background, personality traits, and the intervention were analyzed to assess whether adolescents’ migration or non-migration backgrounds moderated the relationship between personality traits and the effectiveness of the intervention on ethnic-racial identity development. For ethnic-racial identity exploration (participation), no significant three-way interaction effects were found between migration background, personality traits, and the intervention. This included openness (*b* = −0.006, *p* = 0.81), conscientiousness (*b* = −0.003, *p* = 0.86), extraversion (*b* = 0.001, *p* = 0.97), agreeableness (*b* = −0.033, *p* = 0.14), and neuroticism (*b* = −0.000, *p* = 0.98). Similarly, no significant three-way interaction was observed for the meta-traits plasticity (*b* = −0.006, *p* = 0.81) or stability (*b* = −0.015, *p* = 0.53).

In terms of ethnic-racial identity exploration (search), no significant three-way interaction effects were found between migration background, personality traits, and the intervention. Results for the five traits were non-significant: openness (*b* = 0.007, *p* = 0.68), conscientiousness (*b* = −0.010, *p* = 0.46), extraversion (*b* = −0.019, *p* = 0.17), agreeableness (*b* = −0.014, *p* = 0.44), and neuroticism (*b* = 0.003, *p* = 0.78). Likewise, neither plasticity (*b* = −0.017, *p* = 0.36) nor stability (*b* = −0.018, *p* = 0.37) yielded significant three-way interaction effects. For ethnic-racial identity resolution, no significant three-way interaction effects were found for any of the personality traits: openness (*b* = 0.021, *p* = 0.23), conscientiousness (*b* = 0.011, *p* = 0.42), extraversion (*b* = 0.005, *p* = 0.70), agreeableness (*b* = −0.019, *p* = 0.29), and neuroticism (*b* = −0.001, *p* = 0.91). Similarly, no significant interactions were found for the meta-traits plasticity (*b* = 0.014, *p* = 0.45) or stability (*b* = 0.001, *p* = 0.96). While three-way interaction effects with migration backgrounds were non-significant, the slope impact and effect sizes of these interactions were sometimes large (Table 2).

**Table 2:** Latent growth model estimates for ethnic-racial identity exploration (participation)

Model	Intercept (*i*)	SE (*i*)	Standardized β (*i*)	Slope (*s*)	SE (*s*)	Standardized β (*s*)	CFI	RMSEA	BIC
Unconditional Growth	2.401***	0.039		0.020***	0.003	0.628	1.000	0.000	3002
Experiment vs. Control	−0.210**	0.079	−0.152	0.019***	0.005	0.272	0.998	0.020	3027
Openness Model							1.000	0.000	2608
Openness	0.257*	0.125	0.207	−0.001	0.009	−0.019			
Interaction	−0.130	0.161	−0.083	0.006	0.011	0.074			
Gender				−0.014	0.025	−0.084	0.988	0.031	2654
Migration background				−0.006	0.023	−0.056	0.995	0.023	2506
Conscientiousness Model							1.000	0.000	2656
Conscientiousness	0.176*	0.088	0.166	−0.001	0.006	−0.016			
Interaction	0.046	0.129	0.032	0.001	0.009	0.012			
Gender				−0.028	0.021	−0.202	0.974	0.046	2657
Migration background				−0.003	0.018	−0.033	1.000	0.000	2516
Extraversion Model							0.992	0.033	2662
Extraversion	0.079	0.096	0.078	−0.006	0.006	−0.116			
Interaction	−0.168*	0.084	−0.126	0.027***	0.008	0.402			
Gender				−0.011	0.020	−0.079	0.987	0.033	2659
Migration background				0.001	0.017	0.008	0.996	0.021	2519
Agreeableness Model							0.971	0.067	2667
Agreeableness	−0.022	0.104	−0.018	0.009	0.007	0.147			
Interaction	−0.042	0.150	−0.026	0.001	0.011	0.007			
Gender				−0.051*	0.023	−0.312	0.964	0.053	2666
Migration background				−0.033	0.023	−0.320	0.973	0.053	2517
Neuroticism Model							0.984	0.051	2658
Neuroticism	−0.014	0.081	−0.017	−0.002	0.005	−0.054			
Interaction	−0.289	0.114	−0.245	0.007	0.008	0.130			
Gender				0.019	0.017	0.207	0.979	0.042	2658
Migration background				−0.000	0.016	−0.006	0.977	0.049	2519
Stability Model							0.994	0.030	2659
Stability	0.109	0.126	0.081	0.005	0.007	0.080			
Interaction	0.273	0.179	0.150	−0.006	0.011	−0.065			
Gender				−0.054*	0.025	−0.308	0.984	0.036	2657
Migration background				−0.015	0.023	−0.131	0.991	0.030	2517
Plasticity Model							0.996	0.025	2662
Plasticity	0.199	0.124	0.147	−0.006	0.008	−0.084			
Interaction	−0.192*	0.083	−0.145	0.024*	0.010	0.360			
Gender				−0.009	0.028	−0.044	0.988	0.031	2660
Migration background				−0.006	0.024	−0.050	0.999	0.011	2518

The table reports unstandardized and standardized estimates for intercept (i) and slope (s), with corresponding standard errors (SE). Model fit indices include Comparative Fit Index (CFI), Root Mean Square Error of Approximation (RMSEA), and Bayesian Information Criterion (BIC). Interaction terms represent the moderating effect of personality traits on the intervention’s impact on ethnic-racial identity exploration trajectories. Gender and Migration background represent the three-way interaction models

*p < 0.05, **p < 0.01. ***p < 0.001

In summary, migration background did not significantly moderate the relationship between personality traits or meta-traits and the intervention’s effects on ethnic-racial identity development across the three ethnic-racial identity dimensions: exploration (participation), exploration (search), and resolution. For full details regarding non-significant two- and three-way model estimates, please see the supplementary material available on the Open Science Framework: ethnic-racial identity exploration (search) (https://osf.io/v9a5u), and ethnic-racial identity resolution (https://osf.io/v28bw).

### Gender as a Moderator of Personality Trait Influence on Intervention Efficacy

Three-way interaction effects between gender, personality traits, and the intervention were examined to assess whether gender moderated the relationship between personality traits and the intervention’s impact on ethnic-racial identity development. For ethnic-racial identity exploration (participation), gender was found to significantly moderate the association between agreeableness and the intervention effect (*b* = −0.051, *p* = 0.023, *β* = −0.312). These results indicate that agreeableness mattered more for boys’ responsiveness to the intervention than it did for girls’. Model fit indices for this interaction model were strong (*CFI* = 0.964; *RMSEA* = 0.053; *BIC* = 2666). Similarly, a significant three-way interaction was found between gender, the meta-trait stability, and the intervention effect (*b* = −0.054, *p* = 0.021, *β* = −0.304), with fit indices indicating a good fit for the model (*CFI* = 0.957; *RMSEA* = 0.054; *BIC* = 2669). No significant gender-moderated three-way interaction effects were found for openness (*b* = −0.014, *p* = 0.58), conscientiousness (*b* = −0.028*, p* = 0.19), extraversion (*b* = −0.011, *p* = 0.59), or neuroticism (*b* = 0.019, *p* = 0.28). Likewise, the meta-trait plasticity did not show any significant gender-moderated three-way interaction with the intervention effect (*b* = −0.009, *p* = 0.76).

For ethnic-racial identity exploration (search), no significant three-way interaction effects involving gender were found for any of the personality traits or meta-traits: openness (*b* = 0.021, *p* = 0.27), conscientiousness (*b* = 0.015, *p* = 0.36), extraversion (*b* = 0.026, *p* = 0.12), agreeableness (*b* = 0.002, *p* = 0.79), neuroticism (*b* = −0.007, *p* = 0.68), stability (*b* = 0.018, *p* = 0.47), and plasticity (*b* = 0.038, *p* = 0.06). Similarly, no significant interactions were found between gender and any personality traits or structures in predicting ethnic-racial identity resolution: openness (*b* = −0.002, *p* = 0.92), conscientiousness (*b* = −0.014, *p* = 0.37), extraversion (*b* = 0.002, *p* = 0.93), agreeableness (*b* = −0.029, p = 0.10), neuroticism (*b* = −0.010, *p* = 0.49), stability (*b* = −0.015, *p* = 0.43), and plasticity (*b* = 0.007, *p* = 0.75).

**Fig. 4:**
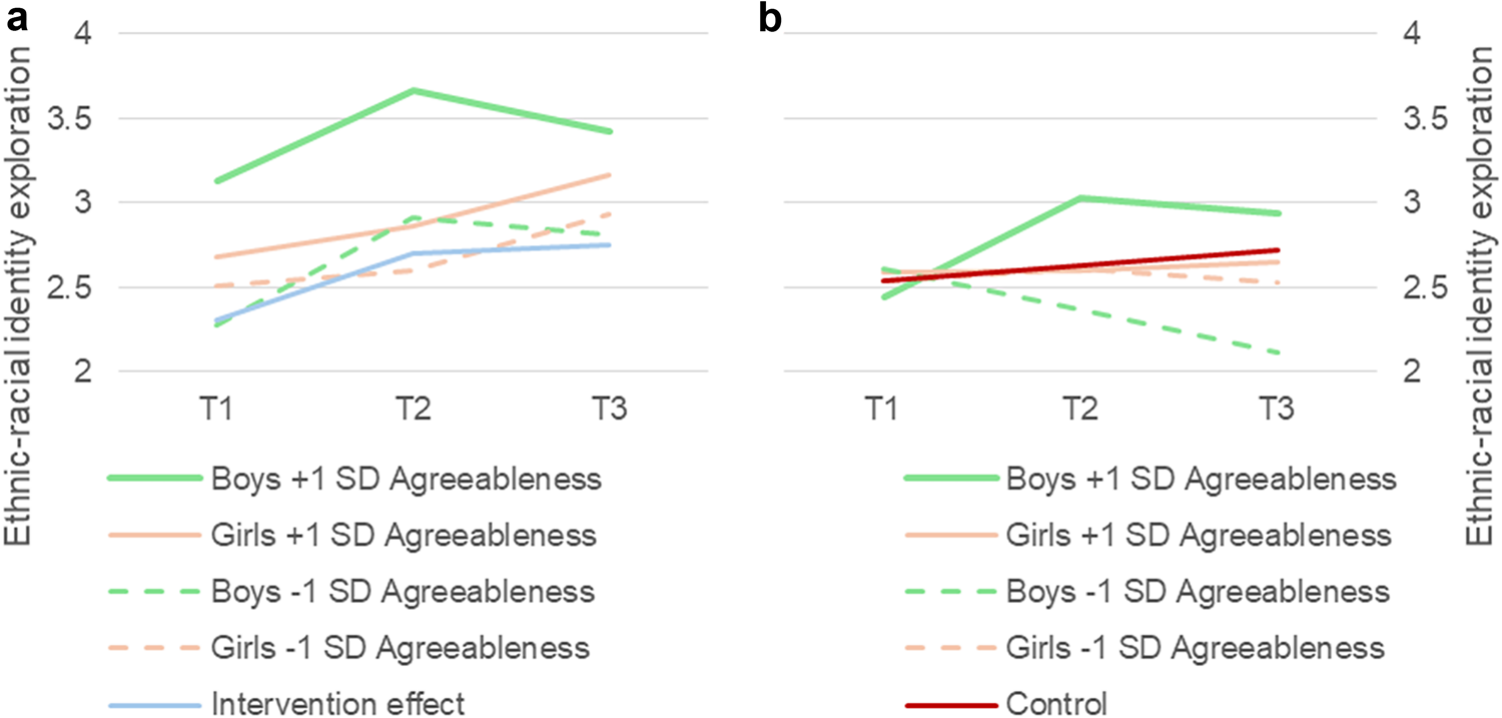
**a** and **b**. Three-way interaction effects of agreeableness on ethnic-racial identity exploration trajectories within the intervention group (Fig. 4a) as well as the control group (Fig. 4b). Gender included as a predictor in the models. Raw means at each time point are displayed for participants at ± 1 SD of agreeableness. T1 = baseline, T2 = 12 weeks, T3 = 16 weeks. See Table 2 for standard errors of trajectories

**Fig. 5:**
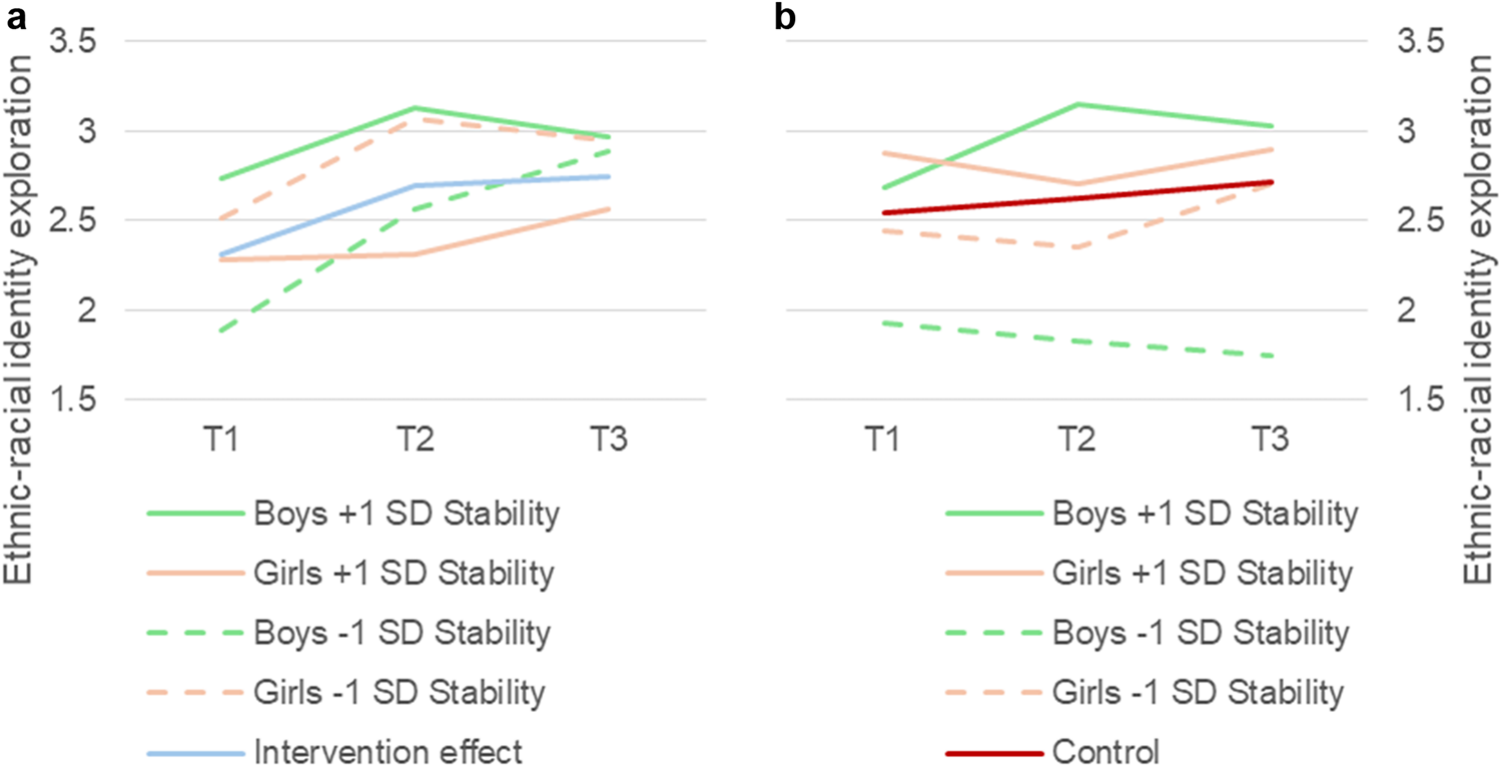
**a** and **b**. Three-way interaction effects of stability on ethnic-racial identity exploration trajectories within the intervention group (Fig. 5a) as well as the control group (Fig. 5b). Gender included as a predictor in the models. Raw means at each time point are displayed for participants at ± 1 SD of stability. T1 = baseline, T2 = 12 weeks, T3 = 16 weeks. See Table 2 for standard errors of trajectories

In summary, the patterns of ethnic-racial identity exploration varied between genders across different levels of agreeableness and stability. When agreeableness or stability are included in the three-way interaction models, boys initially increase in ethnic-racial identity exploration between T1 and T2, followed by a deteriorating effect between T2 and T3. Boys high in agreeableness reported significantly higher ethnic-racial identity exploration scores throughout, while boys low in stability reported lower initial scores. Girls reveal more stable growth between T1 and T3, an interaction effect that is more clear for the trait agreeableness (Fig. 4a, b) than for the meta-trait stability (Fig. 5a, b). These results indicate that boys’ responsiveness to the intervention depends more on their levels of agreeableness and stability, as compared to girls’ responsiveness.

### Supplementary Analyses

All personality traits and interaction effects were initially analyzed in a single model, however, subsequent analyses were performed with personality traits and meta-traits separated into different models (the output from these separated models are also what is reported in the Results section). This was decided for three reasons: (1) personality traits are distinct and well-validated constructs (Zell & Lesick, 2022); (2) multi-layer interaction effects can create convoluted models, especially with multiple independent variables; and (3) every interaction effect also includes all variables of interest, which quickly leads to issues with multicollinearity (Schwarz et al., 2014). Although personality traits were run in separate models, significant interaction effects between traits, gender, and migration background may still exist but possibly be obscured by inflated standard errors due to multicollinearity (Tarka, 2018). The decision to include traits and meta-traits in separate models resulted in a slightly better model fit and no difference in significant findings or directions of change: model fit indices for the models in which each personality trait was included separately were typically strong (e.g. extraversion/exploration (participation): *CFI* = 0.992; *RMSEA* = 0.035; *BIC* = 2662). In contrast, model fit indices for the large models in which all personality traits and their respective interaction effects were included were worse fitting and showed less favorable model complexity trade-offs (e.g. all traits/exploration (participation): *CFI* = 0.944; *RMSEA* = 0.059; *BIC* = 2701).

To explore the possibility of non-linear growth within the models, quadratic growth models were also attempted, but more often than not these models would not converge. This is likely due to the known challenges of estimating non-linear trends with only three time points (Duncan et al., 2013), an issue furthered by the inclusion of interaction effects, which increase model complexity and possible overparameterization. Given these challenges and the limits of having only three measurement points, linear growth models were employed and reported for all analyses in the manuscript.

## Discussion

Understanding which adolescents benefit most (or least) from interventions supporting identity development is important for improving their effectiveness. While the Identity Project intervention led to increases in both ethnic-racial identity exploration and resolution in the Swedish sociocultural context (Abdullahi et al., 2024), little is known about adolescents’ individual differences, such as personality traits, and how they shape responsiveness to the intervention. To address this gap, the role of personality traits and meta-traits in moderating intervention effectiveness was examined. Adolescents who scored higher in extraversion and plasticity also demonstrated greater positive change in ethnic-racial identity exploration following the intervention, indicating that certain traits have the potential to enhance exploration processes in identity-based interventions. Further moderating analyses also revealed that the personality trait agreeableness as well as the meta-trait stability had gender-specific effects, influencing outcomes more for boys than for girls. Other personality traits did not moderate the intervention’s efficacy, nor did participants’ migration backgrounds moderate the effectiveness of the intervention, suggesting consistency in responses across diverse groups. The findings suggest that both personality traits and meta-traits, particularly extraversion and plasticity, play a significant role in adolescents’ responsiveness to interventions targeting ethnic-racial identity developments.

### Personality Traits and Intervention Response

As implied by the results of this study, adolescents with higher levels of extraversion and plasticity may be particularly well suited for interventions like the Identity Project. On the contrary, adolescents who participated in the intervention and reported lower levels of extraversion or plasticity did not significantly differ from those in the control group. These findings suggest that the differences in ethnic-racial identity exploration based on personality traits are not merely a matter of varying degrees of effectiveness; rather, adolescents with lower levels of extraversion and plasticity appeared to derive little to no benefit from the intervention at all. One way to understand these findings can be through the personality traits themselves. Extraversion, a trait often linked to an increased degree of social activity (Wilt & Revelle, 2015), is in this case likely also associated with greater classroom interaction with peers. Such peer interactions are important aspects of the program curriculum (https://osf.io/dknhs), which includes both structured group activities and classroom discussions as well as interviews with people outside school. While students could technically choose not to fully engage in these activities, the intervention was designed and delivered with these social components. For adolescents high in extraversion, the activities may thus have enhanced social interaction and exploration, whereas less extraverted participants might have found the same components less stimulating or even uncomfortable. It is possible that these social and interactive elements may explain some of the differences in the adolescents’ responsiveness, leading to weaker intervention effects. Notably, it was the participatory aspects of ethnic-racial identity exploration, rather than cognitive search processes, that revealed significant interaction effects. One could interpret this as the intervention’s socially engaging components having played a more important role in the personality-based differences in responsiveness, but also that there simply may not have been enough change in the search-related dependent variable. The intervention in focus does seem to primarily have an effect on participatory aspects of ethnic-racial identity exploration (Abdullahi et al., 2024). As little is also known about the effectiveness of the different lessons and parts of the curriculum, it is possible that some more socially demanding parts of the intervention held much of the effect; an effect that was therefore missed by some participants.

Another way to understand the findings regarding extraversion and intervention effect is through the Cybernetic Big Five Theory (DeYoung, 2015). This theory views personality traits as parameters within a goal-directed, cybernetic system, in which the brain continuously evaluates the reward value of different stimuli and uses this information to guide behavior. Within this framework, extraversion is characterized by a heightened sensitivity to social and other rewarding stimuli, motivating approach-oriented behaviors and promoting goals such as peer socialization. In line with previous research, a heightened sensitivity to rewards and goal-oriented behaviors has actually been found to be especially prominent in individuals high in extraversion (Smillie, 2013). Therefore, extraverted adolescents, driven by a higher sensitivity to social rewards, may have been more inclined to seek out the interactive opportunities of the program, leading to more positive responses to the intervention. On the contrary, individuals lower in extraversion may have perceived the social components of the curriculum as less rewarding, which may also explain their lessened response to the intervention.

The second trait that moderated intervention effectiveness, the meta-trait plasticity, consists of both extraversion and openness and is often associated with different forms of adaptability or exploration. While extraversion reflects a more behavioral aspect of exploration that relates to goals, openness reflects a more cognitive aspect of exploration, such as engaging with and reflecting on new information (DeYoung et al., 2012). The results of this study indicate that higher levels of plasticity promoted greater identity exploration in the intervention, and thus also greater intervention effectiveness. One possible reason for this could be that adolescents high in plasticity may simply feel more comfortable in the self-reflective and cognitively explorative tasks of the intervention, making them more responsive. Or, as plasticity can also be understood as an individual’s tendency for both cognitive and behavioral exploration (DeYoung, 2015), plasticity may also promote greater gains in terms of ethnic-racial identity exploration when both these aspects are tapped.

The gendered differences when stability and agreeableness were modeled also provide important information. These results suggest that some boys in the classrooms are likely to see a tapering-off effect in ethnic-racial identity exploration following the intervention as compared to girls, who revealed more continuously positive trajectories. Interestingly enough, boys with a high score on the trait agreeableness also reported significantly higher scores on ethnic-racial identity exploration throughout, as compared to boys with a low scores on agreeableness. On the other hand, boys in the control group with low scores on stability also reported very low scores on ethnic-racial identity exploration. With scores on the traits agreeableness and the meta-trait stability seemingly being more important when it comes to intervention effect for boys than for girls, it is plausible that boys are operating within a more rigid, self-regulating system. From a cybernetic perspective, their behavioral control mechanisms may be more inclined to maintain established patterns rather than incorporating new explorations prompted by the intervention.

The different operationalizations of personality used in this study (Big Five personality traits and higher-order meta-traits) also offered some valuable insights regarding intervention responsiveness. While the Big Five model provided more detailed information on personality traits like extraversion and agreeableness, the meta-traits, on the other hand – plasticity and stability – captured broader tendencies. With individual traits (such as openness) not being significantly associated with intervention effect but plasticity being so, it is important to continue considering these different meta-levels of personality. In acknowledging how a five-factor (McCrae & Costa, 1987) or even ten-factor personality model (DeYoung et al., 2016) can provide detailed information, it is also important to acknowledge how the shared variance behind these traits may hide significant, higher-order associations (DeYoung, 2006). As demonstrated by the results of this study, looking at personality traits and structures in multiple ways and at multiple levels may help researchers better understand differences in responsiveness to an intervention such as the Identity Project. Building on these trait perspectives, extraversion and plasticity reflect an individual’s exploration and openness to change, both cognitively and behaviorally. For individuals with lower scores on these traits, interventions that heavily rely on both behavioral and reflective exploration may not resonate as strongly, reducing their effectiveness. This study’s findings thus underscore the importance of considering individual differences in personality traits when designing interventions. Interventions that accommodate a broader range of personality profiles, might indeed be more effective.

### Differential Susceptibility and Intervention Design

The impact of personality traits on intervention effectiveness seen in this study highlights the concept of “differential susceptibility”, suggesting that not all adolescents will respond equally to what is provided (Belsky & Pluess, 2009). While prior studies in related fields demonstrate varied responses to interventions based on different personality traits (de Vibe et al., 2015), the present study indicates that the Identity Project’s classroom-delivered approach may particularly reach adolescents with high social and adaptable tendencies. These findings – suggesting that differential susceptibility is relevant for identity-related interventions – align with previous research on environmental sensitivity (Pluess, 2015). Adolescents with higher environmental sensitivity are also more likely to benefit from interventions with strong identity and social components, as seen in a previous study that also examined the Identity Project intervention (Ceccon et al., 2023). Opposite to the adolescents who were more sensitive to – and benefited more from – the intervention are, then, those who were more introverted or had higher levels of stability. While the results of this study do not reveal whether or not these adolescents might prefer other intervention formats, its findings indicate the need for adaptations that include these adolescents in better ways. Such adaptations could make interventions more effective in supporting adolescents with a broad range of personalities.

Practical adaptations to encompass these differences in effectiveness could involve providing smaller group sessions or one-on-one opportunities. For instance, incorporating more flexible sessions where students can opt for individual, self-paced tasks may help adolescents who prefer quieter, more reflective settings. This could also be extended to homework tasks, in which options could be offered regarding, for example, interviewing a member of one’s ethnic-racial community. Activities that involve journaling or self-reflection prompts are already in the Identity Project curriculum, but could perhaps be further tapped in some of the more social interaction-dependent sessions. One could also consider developing alternative facilitator approaches to encourage more identity exploration across different personality profiles. For example, facilitators could incorporate differently structured discussion formats in which students are encouraged to gather their thoughts before sharing in smaller constructed groups, or use more “warm-up” activities to help adolescents ease into social interactions (one such activity is part of the first session). This also raises the question of how much, and in what way, moderators should try to engage participants in different social interactions, and whether such approaches should differ between individuals or classrooms. Previous research has, in fact, revealed significant differences in classroom preferences for items such as “engaging in discussions with other students” between extraverted and introverted adolescents (Murphy et al., 2017). These aspects can also be applied to the larger contextual settings, in this case Sweden, likely having its own impact on the way in which adolescents find it easy, or hard, to engage in topics surrounding for example ethnicity. Given Sweden’s emphasis on colorblind ideals in public discourse and records (Hübinette et al., 2023), one could argue that discussions on ethnic-racial identity may sometimes be met with discomfort or even reluctance. Adolescents could therefore vary in their willingness to engage in the contents of the intervention, possibly dependent on how the topics within are currently being discussed in their lives. Looking at the Swedish sociocultural context perspective, adolescents’ ethnic-racial identity development also takes place in a society that is both multicultural and increasingly polarized in, for example: attitudes towards migration (European Social Survey European Research Infrastructure, 2023). Adolescents’ engagement with ethnic-racial identity interventions may be impacted by their lived experiences of either inclusion or marginalization. For some adolescents, interventions like the Identity Project likely offer an opportunity to explore and make sense of their ethnic-racial identities, whereas other adolescents may approach identity exploration with hesitation. Future research could do well in examining, in more detail, how these sociocultural aspects influence intervention responsiveness and ethnic-racial identity development over time.

To also address the gendered differences in adolescents’ responsiveness, for example adolescents with high scores on stability, incorporating more goal-setting activities into identity-related interventions may be advantageous. Such approaches could provide slightly clearer “targets” (and more approachable outcomes), possibly appealing to those who prefer structure or gradual progress. With potential rewards of identity development made more explicit and attainable, these adolescents might then see the value in exploring their identities long-term, and hopefully also increase their long-term responsiveness to the intervention. By acknowledging that not all adolescents benefit equally from the intervention, one can consider strategies like these as a starting point for making identity-related interventions more responsive to broader ranges of participants. While the adaptations discussed above are only a few examples, they highlight how interventions might be refined to better reach those who prefer structure, clear goals, or less socially focused activities. Moving forward, researchers should keep the results of this study in mind when designing new or adapting current interventions, with the goal of better reaching adolescents with different personality profiles and thereby potentially increasing overall responsiveness.

### Limitations

Several limitations of this study should be noted, starting with the fact that the adolescents who participated in the intervention were in their first year of high school. This may have influenced social dynamics compared to previous intervention samples in which participants were already familiar with one another. It is possible that this dynamic of “being new” made less extraverted adolescents even less inclined to socially engage with each other. As the intervention was also facilitated during the COVID-19 pandemic, this may have further limited social interaction within the classrooms. While the Swedish schools included in the study were kept open during the pandemic, it is still possible that factors such as social distancing impacted the study’s results. The time-frame of the measurements is another potential limitation to consider. With more time between measurements, it is possible that any delayed growth in ethnic-racial identity development (and also interactions with personality traits) could have emerged (Syed & Azmitia, 2009). On the other hand, the decision to use a medium-length temporal constraint was motivated and deemed reasonable, as this also limits the number of confounding variables. Furthermore, some subscales of the Big Five Inventory scale demonstrated slightly lower than optimal internal consistency. The instrument was originally validated for individuals aged 18 to 80 (Zakrisson, 2010) and has been previously used (Wängqvist et al., 2015) – but not been extensively trialed – with adolescents in Sweden. While this could have influenced the reliability and interpretation of the personality measures in the current sample, the Big Five hierarchy has repeatedly demonstrated cross-cultural and age-related measurement invariance (Hughes et al., 2021), strengthening that the trait structure remains valid also for adolescents within the Swedish population.

Statistical multicollinearity also proved to be an issue in some of the models, as latent variables sometimes included the same data points (especially within the three-way interaction models). In terms of output, the effect sizes reported in Table 2 of the Results section does provide information about the strength of associations, but should also be interpreted with caution. Latent growth curve models are influenced by model complexity, sample size, and measurement error, something especially relevant for interaction effects, in which large effect sizes may not always reflect stable patterns. Interpreting effect sizes for the three-way interaction effects, even standardized ones, is also inherently challenging. Another statistical limitation of this study is that the design used herein only included three time points, which severely limited the ability to model non-linear growth patterns such as quadratic change. The somewhat large standard errors that were observed, primarily in the three-way interaction effects containing migration background, might potentially also mask some significant findings. Future research could expand on these findings by examining the moderating effect of more specific ethnoracial groups within their samples. While migration background served to capture a set of adolescents who likely share somewhat similar experiences of being minoritized, a more targeted approach—such as examining differences in moderating effects across specific ethnoracial groups—may reveal meaningful distinctions among these groups. This, in turn, might offer clearer insight into how different experiences and contexts shape adolescents’ responses to identity-related interventions. Additionally, the Identity Project intervention has so far only been examined as a unified whole, meaning that researchers are unable to identify which specific lessons or components that have more/less impact. This limitation restricts the understanding of how particular elements drive responsiveness or shape ethnic-racial identity developmental outcomes. Lastly, related but distinct intervention programs that promote adolescents’ identity development—but vary in content or format— may produce different patterns of interaction regarding personality traits and responsiveness. In considering the generalizability of this study’s findings, it may be useful to compare the Identity Project with other identity interventions going forward.

## Conclusion

Personality traits are known to influence various psychosocial outcomes, yet their role in moderating the effectiveness of interventions supporting identity development remains underexplored. This study investigated whether personality traits, the Big Five model and higher-order meta-traits, impacted the outcomes of the Identity Project, a school-based intervention aimed at promoting ethnic-racial identity exploration among adolescents. While the Identity Project did lead to more ethnic-racial identity exploration for the intervention group compared to the control group, the study’s findings reveal that not all adolescents benefited equally. Specifically, those who were lower in the trait extraversion or the meta-trait plasticity appeared to benefit less from the intervention, suggesting that personality indeed impact how adolescents benefit from identity-based interventions. While gender moderated the relationship between agreeableness, stability, and intervention effect, several other traits – as well as participants’ migration backgrounds – did not impact the effectiveness of the intervention. By providing more knowledge about the role that personality traits and meta-traits play in intervention efficacy, the study findings can help guide researchers toward more effective interventions. By adapting identity-related interventions accordingly, so as to also benefit the less extraverted and plastic adolescents, their reach can be broadened and their impact increased.
